# Pancreatic autoimmunity: An unknown etiology on patients with assisted reproductive techniques (ART)-recurrent reproductive failure

**DOI:** 10.1371/journal.pone.0203446

**Published:** 2018-10-22

**Authors:** Diana Alecsandru, Ana Barrio, Victor Andia, Edgar Cruz, Pilar Aparicio, Jose Serna, Maria Cruz, Antonio Pellicer, Juan Antonio Garcia-Velasco

**Affiliations:** 1 Department of Immunology and Department of Reproductive Endocrinology and Infertility, Valentian Infertility Institute (IVI), IVI RMA, Madrid, Madrid, Spain; 2 Department of Endocrinology and Diabetes, Gregorio Marañon University Hospital, Madrid; 3 Valencian Infertility Institute (IVI), IVI RMA, Madrid, Spain; 4 Valencian Infertility Institute (IVI), IVI RMA, Zaragoza, Spain; 5 Valencian Infertility Institute (IVI), IVI RMA, Valencia, Spain; 6 Rey Juan Carlos University, Madrid, Spain; Medical University of Vienna, AUSTRIA

## Abstract

Pancreatic Autoimmunity is defined as the presence of autoantibodies and more frequent need for insulin treatment. Affected women presenting recurrent implantation failure (RIF) or recurrent miscarriage (RM) are often misdiagnosed. The objective of thestudy was to describe clinical and metabolic profiles suggestive of Pancreatic Autoimmunity and therapeutic strategy in patients with RIF/RM. We analyzed retrospectively 735 patients, and have identified a subset (N = 20) with similar metabolic characteristics. At the same time, we included a control group (n = 39), with similar demographic characteristics and negative for pancreatic, thyroid or celiac disease autoimmunity. The patients identified with autoimmune metabolic problem (N = 20) had relatives with diabetes mellitus. At 120 minutes after Oral Glucose Tolerance Test (OGTT) low level of insulin secretion (<2 IU/ml) was found in 70% of patients. Glutamic acid decarboxylase 65 (GAD 65) antibodies, with or without other autoantibodies, were positive in80% of patients and anti-IA2 alone were positive I the rest. Since pregestational period, insulin administration was recommended for 10 patients, metformin for 4 patients and exclusively diet control in 5 of them. Significantly increased live bith rates (LBR) per cycle were observed after metabolic control (52%) compared with live birth rate (LBR) after cycles without control (7.5%) (p<0.0001). We noticed 2 cases of pre-eclampsia and 6 low-birth weights. Insulin administration was needed during the pregnancy in 68% of patients and after childbirth in 31.57% of them. In our control group, all of patients (n = 39) underwent ART (53.8% SET and 46.1% DET) with a 50% (SET) and 61.9% (DET) live birth rate (LBR) per cycle. Patients with RIF/RM, normal BMI, low insulin levels after OGTT could benefit from additional metabolic immune testing. A correct diagnosis and treatment could have a positive impact on their reproductive results and live birth rate.

## Introduction

In the last decade, mean age of infertile women undergoing ART has increased. Age is one of the main risk factors for disorders like functional glucose impairment or hypertension. In the clinical work up of the patients, only fasting glucose levels are included among metabolic tests. During ART, the “silent” metabolic disorders and their impact over reproductive outcome has been less studied.

Women suffering from recurrent implantation failure (RIF) or recurrent miscarriage (RM) may undergo different tests trying to understand their poor reproductive outcome. Metabolic routine screening for all infertile patients is not recommended, but a tailored approach may be needed for some subsets of patients that could improve their reproductive outcome.

Diabetes is a complex disease and the classification into Type 1 and Type 2 does not include all metabolic disorders related with impaired insulin secretion or action. Type 1 diabetes is an autoimmune disease characterized by immunological pancreatic attack by autoreactive T cells and auto-antibodies with severe loss of insulin secretion. Around 5–14% of patients classified with Type 2 diabetes have diabetes-associated autoantibodies [1], [2], [3]. The term latent autoimmune diabetes in adults (LADA) has been introduced [4] for this autoimmune diabetes characterized by adult onset, presence of diabetes associated autoantibodies and more frequent need for insulin treatment than patients with classical type 2 diabetes. LADA is the most prevalent form of adult-onset autoimmune diabetes and probably the most prevalent form of autoimmune diabetes in general [3]. A high frequency of thyroid and gastric autoimmunity among LADA patients and positive HLA-DR3 and DR4 has been described [5] showing a genetic association among autoimmune endocrine diseases.

In reproductive field, most of the tests to detect functional glucose impairment are used during pregnancy and less is known about its usefulness in the preconception period even more in assisted reproductive treatment (ART). Gestational diabetes mellitus affect 2–17% of pregnancies [6] and is defined as “glucose intolerance with onset or first recognition during pregnancy”. It has been shown that 15–30% of women with gestational diabetes have “pregestational” diabetes and the presence of diabetes-associated autoantibodies was highly variable depending on the nature of antibodies and population studied [7], [8], [9].

Our objective was to describe metabolic and clinical profiles suggestive of pancreatic autoimmunity/LADA and therapeutic strategy in patients with recurrent reproductive failure after ART.

## Material and methods

Between June to December 2015, a total of 735 patients with RM and RIF recruited from the Instituto Valenciano de Infertilidad (IVI) Clinics (Madrid, Valencia and Zaragoza -Spain) have been studied. Patients with a poor reproductive outcome have been referred to our Reproductive Immunology Department (10.4% of total of ART–both IVF and oocyte donation (OD). Institutional Review Board approval was obtained for this study from our reference hospital (Puerta de Hierro Hospital, Madrid, Spain) (reference number: 1506-MAD-046-DA).

### Study population

From a total of 735 studied patients we have identified a subset of patients (N = 20; 2.72%) with similar metabolic feature and poor reproductive outcome. From this, 8 had history of RM (2–5 unexplained miscarriages) and 12 suffered RIF (>3 failed IVF cycles with good quality embryos or > 2 failed donor oocyte cycles). This subset of patients had a total of 63 cycles (63.15% own oocytes and 36.84% donor oocytes): in fact, 40 ET cycles (57.5% single embryo transfer (SET), % 42.5% double embryo transfer (DET)) before the metabolic diagnosis and treatment and 23 (80% SET and 20% DET) after diagnosis and pre-conceptional control. Before undergoing immune study, the patients and their partners had a full fertility screening. This included complete clinical history, physical examination, viral serology, hormonal analysis including AMH, thyroid-stimulating hormone (TSH), thyroxin (T4), prolactin, estrogen and progesterone, pelvic ultrasound, male seminogram and FISH analysis of the sperm. Genetic evaluation included karyotype of both parents and tests for inherited thrombophilic disorders (factor V Leiden, prothrombin G20210A mutation, serum homocysteine, and deficiencies of the anticoagulant protein C, protein S, and antithrombin III).

As part of our immunological investigations, we performed metabolic and immunological tests. Special focus in patients with first and second-degree relatives affected with type 1 and 2 DM and LADA. Metabolic tests included: fasting plasma glucose, fasting plasma insulin, insulin and glucose levels before and after OGTT (Oral Glucose Tolerance Test) 75g (60 and 120 minutes) and glycated hemoglobin (HBA1c) (Conway et al.2014, Kahaly, GJ et al 2016). Patients with TSH>2.5 mIU/L were tested for thyroid peroxidase antibodies (TPO Abs), anti-thyroglobulin antibodies (TG Abs). Patients with impaired OGTT response have been derived to the Endocrinologist and the immune tests have been extended for diabetes-associated autoantibodies (DAA) (antiGAD65, associated antigen-IA2, anti-Insulin antibodies) (Radioimmunoassay KIPM2070 anti GAD 65 Kit, radioimmunoassay KIPM2035 anti insulin Kit, radioimmunoassay KIPM2050 anti IA2 Kit). Patient with positive anti-TPO /TG Abs and DAA were tested for B12 vitamin level and celiac disease Abs (anti-tissue transglutaminase tTG and anti-deamidated gliadin peptide Abs) [10], [5]. The DAA were tested before, during and after pregnancy and their positive values were confirmed. Immunological screening included measurement of anticardiolipin and anti-beta-2-glycoprotein (IgG or IgM) antibodies (Orgentec Diagnostika Gmbh, Mainz, Germany) and lupus anticoagulant.

The specific ART, i.e. IVF with either self or donor oocytes with or without ICSI, was individualized for each couple. The ETs cycles before and after pancreatic autoimmunity diagnosis and treatment were made with the same fertility treatment (63.15% self oocytes and 36.84% donor oocytes).

## Statistics

Categorical and continuous variables were expressed as proportions and means with 95% confidence intervals (95%CI), respectively. Odds ratio (OR) and 95%CI were also calculated for the ca- tegorical variables. Categorical data were compared using the Chi-square analysis and a Fisher’s exact test, where appropriate. A p value less than 0.05 was considered as significant. Statistical analysis was performed using the Statistical Package for Social Sciences, version 17 (SPSS Inc., Chicago, IL, USA).

## Results

Women with history of RM (N = 8) and RIF (N = 12) and their ET cycles before (N = 40, 57.5% SET, 42.5% DET) and after (N = 23, 80% SET and 20% DET) diagnosis and pre-conceptional control were evaluated. The most striking finding of this subgroup of patients is that they had a very low LBR/cycle (7.5%), before metabolic study, that is well below of LBR/cycle reported in our clinic for a similar age group (31.9% own oocytes cycles; 41.5% donor oocytes cycles) (https//:ivi.es).

The mean age of this subset of patients was 37.7 years and their mean BMI (body mass index) was 21.5 kg/m2. All patients had 1^st^ and/or 2^nd^ degree relatives with diabetes mellitus or autoimmune disorders: 3 of them had family history of autoimmune thyroid disease, 2 systemic lupus erythematosus (SLE) and 1 with Sjögren syndrome. Seventeen patients (85%) had autoimmune thyroid disorder with positive TPO Abs.

All patients presented normal HbA1c. At 120 minutes after OGTT (Oral Glucose Tolerance Test) low level of insulin secretion was found in 14 patients (70%), high levels (>60 IU/ml) in 4 (20%), and normal (<60 IU/ml) level in 2 (10%). GAD 65 antibodies (Abs) were positive with high titers in 16 of patients (80%), IA2 Abs in 6 (31.5%) (2 GAD65+IA2 Abs) and Insulin Abs in 4 (21%) (GAD65+Insulin Abs). Patients with very low level of insulin (<2mIU/l) had positive GAD 65Abs (14 patients). All patients were diagnosed as pancreatic autoimmunity associated disorder and have been derived to the Endocrinologist. Table 1.

**Table 1:** Baseline characteristics between groups.

	Study group (n = 20)	Control group (n = 39)	p
Age (years)	37.3 ± 3.1	39.0 ± 3.7	0.104
Basal glucose (mg/dl)	86.0 ± 10.0	83.8 ± 6.9	0.343
HbA1c	5.1%	4.9%	0.091
Basal insulin (UI/mL)	6.6 ± 4.3	8.2 ± 3.9	0.175
Insulin 120 min. (UI/mL)	30.2 ± 6.9	31.5 ± 9.6	0.809

One of patients dropped out and discontinued reproductive treatment. After pancreatic autoimmunity associated disorder diagnosis and treatment, 19 patients underwent ART (80% SET and 20% DET) with the same source of oocytes as previously done (63.15% self oocytes and 36.84% donor oocytes). Insulin administration was recommended for 10 patients (low insulin after OGTT and low fasting C-peptide), metformin for 4 (insulin resistance feature) and diet control in 5 of them since pre-concepcional period. We have observed a significantly increased live birth rate (LBR) per cycle after metabolic control (52%) compared with LBR/cycle without pancreatic autoimmunity control (7.5%) (p<0.0001) in our cohort. We noticed 2 cases of pre-eclampsia and 6 low-birth weights. Insulin administration was needed during the pregnancy in 68% of patients and after childbirth in 31.6% of them. We compared the reproductive outcome between 2 groups: the study group (N = 20) and control group (N = 39) before the metabolic control and after.

Both groups had similar demographic characteristics. All control group patients had normal response after OGTT and negative pancreatic, thyroid and celiac disease autoimmunity.

In our control group, all patients (n = 39) underwent ART (53.8% SET and 46.1% DET) with a 50% and 61.9% LBR per cycle respectively. After comparing the study group before and after intervention, and the control group, we observed a significantly increased live birth rate (LBR) per cycle between all patients (p<0.005). Table 2.

**Table 2:** Live birth rate per cycle before and after treatment versus control group.

	Before treatment (n = 20)	After treatment (n = 19)	Control group (n = 39)		p
			SET	DET	
Live birth rate per cycle	7,5% (2.2–15)	52% (37.9–62.2)	50% (26.9–73.1)	61,9% (41.1–82.7)	0,005

## Discussion

We have studied the autoimmune-glucose related factor in a subset of patients with reproductive failure (RIF and RM), family history of metabolic disorders, normal BMI, normal fasting plasma glucose and low insulin levels after OGTT. After a precise diagnosis and adequate metabolic control, we have observed a significantly increased live birth rates (LBR) per cycle (52%) compared with LBR/cycle without pancreatic autoimmunity control (7.5%) (p<0.0001).

LADA, the most prevalent form of adult-onset autoimmune diabetes, is characterized by the presence of diabetes, associated pancreatic autoimmunity and more frequent need for insulin treatment than patients with type 2 diabetes. The diabetes associated autoantibodies appear time, even years, before LADA diagnosis. The current preconceptional protocols do not include tests to detect pancreatic autoimmunity and affected women presenting RIF or RM are often misdiagnosed. Before the embryo transfer (ET) the routine tests include only fasting plasma glucose and a normal level is considered as normal metabolic function. Immune or metabolic routine screening for all infertile couples is not advised but a tailored approach is useful for some subsets of patients having “silent” immune or metabolic disorders.

Anti-GAD Antibodies (Abs) can be detected years before the clinical onset of autoimmune diabetes, which indicate a long pre-diabetes autoimmune period. Among women with gestational diabetes 15–30% have “pregestacional” diabetes and the presence of diabetes-associates autoantibodies is highly variable [7], [8], [9] and unknown in patients undergoing to ART. Anti-GAD positivity is associated with high risk HLA haplotypes linked to type 1 DM and also thyroid autoimmunity [11], [12]. HLA DQA1-DQB1 haplotypes were associated with the presence of antiGAD Abs and GAD positivity is coupled to autoimmunity and association between type 1 diabetes or LADA and thyroid autoimmunity [11], [13], [14].

Thyroid autoimmune disorder is the most common autoimmune condition in women of childbearing age (female/male] ratio = 15/1) [15]. TSH determination is included in all pregestational panel tests for all patient underwent to ART and there is a consensus about their management [16]. In contrast, for glycaemic disorders only fasting glucose is tested before starting ART and this determination falls short in some subset of patients. Recently, it has been recommended [17] the use of OGTT in obese women, in lean PCOS women with advanced age (>40 years), (increased demand for ART) as well as in the presence of a personal history of gestational diabetes or family history of type 2 DM [18], [19], but few details are known about the autoimmune diabetes among reproductive age and risk population.

In our subset of patients (2.7% of our RM and RIF cohort) we have observed that patients affected by autoimmune pancreatic disorders, using the routine tests and having normal BMI, HbA1c, fasting glucose and insulin level, could be considered as having a normal metabolic state before ART. But, 85% of them have thyroid autoimmune disease associated with family history of DM (100%) and it is known that the autoimmune thyroid disease and autoimmune diabetes frequently occur in the same individual because of a strong shared genetic susceptibility [20]. We think that patients with RM or RIF of unknown etiology diagnosed with thyroid autoimmune disorders, family history of diabetes and impaired insulin response after OGTT could be considered as a subset of patients, candidates for further specific autoimmune tests to rule out DAA.

In patients with family history of DM, the OGTT could help to detect the insulin secretion impairment even more if those patients have other autoimmune disease. GAD65 Abs is by far the most common autoantibody in adult-onset diabetes [21] and LADA is the most prevalent form of adult-onset autoimmune diabetes and probably the most prevalent form of autoimmune diabetes in general.

The incidence of pancreatic autoimmunity or LADA among women undergoing to ART is unknown (2.72% in our RM and RIF cohort), but it is known that LADA patients have more frequent need for insulin treatment than patients with type 2 diabetes: its early diagnosis and treatment definitely improve their metabolic control and could have a positive impact over their reproductive outcome.

It is generally accepted that autoreactive T-cells are responsible for the inflammatory pancreatic β cells destruction in autoimmune diabetes [22]. The chronic inflammation of pancreatic islets plays a pivotal role in the development of the disease and the immune responses are the millstone in the mechanism of endothelial activation, atherosclerosis plaque development demonstrated by correlation between levels of inflammatory markers and the risk of cardiovascular events in patients with DM [23]. Small studies have described differences in LADA regarding to abnormal DNA methylation in CD4+T cells [24], altered T-regulatory cells [25], NK cells [26].

Fetal protection from rejection of fetal antigen-reactive T cells has been attributed to suppression of T cells proliferation by several pathways as part of maternal immune tolerance. [27]. Maternal-fetal tolerance process is also mediated by CD4 regulatory T cells (Treg) observed by the increase of this cells during the pregnancy in human decidua [28]. In LADA patients, altered Treg cells and a subset of CD4+ cells (CD4+CD28null) show resistance to apoptosis and exert direct cytolytic effects on endothelial cells and proapoptotic effects on smooth muscle cells [29] effects related with the cardiovascular risk in these patients. These mechanisms could interfere with the maternal-fetal tolerance process in pancreatic autoimmunity women and increase the risk of altered early placentation.

The correct management of diabetes has a positive impact on controlling the systemic inflammatory status, feature of the disease. From the reproductive point of view, it could provide a more favorable maternal environment for embryo implantation. In our subset of patients, the reproductive outcome showed a significant improvement after the autoimmune pancreatic diagnosis and treatment, quantified by the increased LBR (52% vs 7.5%) (p<0.0001).

The incidence of pancreatic autoimmunity or LADA among women undergoing to ART is unknown and probably is not so high (2.7% in our RM and RIF cohort); but is quite easy, by clinical and metabolic characteristics described, to identify this subset of patients and recommend a correct treatment with a positive impact in their pre-conception management and reproductive result after ET.

This is the first report about pancreatic autoimmunity-LADA implication on ART, this metabolic disorder could be a risk factor for RM, and RIF now considered as unknown etiology. Being a retrospective study, larger studies are needed to confirm our findings and demonstrate the mechanisms of autoimmune diabetes-immune disorder at site of placentation.

## Conclusions

Adult autoimmune endocrine pancreatic disorders are often misdiagnosed on women undergoing ART. Patients with reproductive failure, 1^st^ and/or 2^nd^ degree relatives with metabolic or autoimmune disorders, normal BMI, low insulin levels after OGTT could benefit from additional immune testing to rule out pancreatic autoimmunity. Serum GAD65 antibodies is by far the most common autoantibody in adult-onset diabetes. An early and pre-conceptional diagnosis and treatment could have a positive impact on their reproductive results and live birth rate.
